# Synaptic bouton properties are tuned to best fit the prevailing firing pattern

**DOI:** 10.3389/fncom.2014.00101

**Published:** 2014-09-09

**Authors:** Markus M. Knodel, Romina Geiger, Lihao Ge, Daniel Bucher, Alfio Grillo, Gabriel Wittum, Christoph M. Schuster, Gillian Queisser

**Affiliations:** ^1^Bernstein Group Detailed Modeling of Signal Processing in Neurons, University of Heidelberg and University of FrankfurtHeidelberg/Frankfurt, Germany; ^2^Department of Simulation and Modeling, Goethe Center for Scientific Computing, University of FrankfurtFrankfurt, Germany; ^3^Bernstein Center for Computational Neuroscience Heidelberg-MannheimHeidelberg/Mannheim, Germany; ^4^Department of Neurobiology, Interdisciplinary Center for Neurosciences, University of HeidelbergHeidelberg, Germany; ^5^Development Unit, European Molecular Biology LaboratoryHeidelberg, Germany; ^6^Department of Mathematical Sciences, Polythecnic of TurinTurin, Italy; ^7^Department of Computational Neuroscience, Goethe Center for Scientific Computing, University of FrankfurtFrankfurt, Germany

**Keywords:** neuromuscular junction, boutons, modeling and simulation, structure-function relationships, morphology, firing pattern, adaption

## Abstract

The morphology of presynaptic specializations can vary greatly ranging from classical single-release-site boutons in the central nervous system to boutons of various sizes harboring multiple vesicle release sites. Multi-release-site boutons can be found in several neural contexts, for example at the neuromuscular junction (NMJ) of body wall muscles of *Drosophila* larvae. These NMJs are built by two motor neurons forming two types of glutamatergic multi-release-site boutons with two typical diameters. However, it is unknown why these distinct nerve terminal configurations are used on the same postsynaptic muscle fiber. To systematically dissect the biophysical properties of these boutons we developed a full three-dimensional model of such boutons, their release sites and transmitter-harboring vesicles and analyzed the local vesicle dynamics of various configurations during stimulation. Here we show that the rate of transmission of a bouton is primarily limited by diffusion-based vesicle movements and that the probability of vesicle release and the size of a bouton affect bouton-performance in distinct temporal domains allowing for an optimal transmission of the neural signals at different time scales. A comparison of our *in silico* simulations with *in vivo* recordings of the natural motor pattern of both neurons revealed that the bouton properties resemble a well-tuned cooperation of the parameters release probability and bouton size, enabling a reliable transmission of the prevailing firing-pattern at diffusion-limited boutons. Our findings indicate that the prevailing firing-pattern of a neuron may determine the physiological and morphological parameters required for its synaptic terminals.

## 1. Introduction

Chemical synapses are specialized compartments of neurons for neuron-to-neuron or neuron-to-muscle communication that exist in various morphological layouts (Rollenhagen and Lubke, 2006) ranging from classical single-release site synapses at cortical neurons (Gulyas, 1993) over intermediate-sized multi-release-site boutons of the calyx of Heldt (Satzler et al., 2002) or the arthropod neuromuscular junctions (NMJs) (Bradacs et al., 1997) to giant multi-release-site terminals at vertebrate NMJs (Dreyer et al., 1973). While the principal processes underlying synaptic transmission and plasticity at these synapses are rather well understood, the role that the various morphological layouts may play in shaping synaptic functions is currently unclear. One reason for this astonishing discrepancy may be the difficulties to systematically control the morphological and physiological configuration of a given synaptic system.

Prominent examples for two highly related but morphologically distinct multi-release-site boutons are the well-characterized glutamatergic synapses at larval NMJs of *Drosophila* (Jan and Jan, 1976; Atwood et al., 1993; Schuster, 2006; Collins and DiAntonio, 2007). Larval body wall muscles of *Drosophila* are typically innervated by two motor neurons of which one forms large spherical and somewhat variable type Ib boutons and a second motor neuron forms smaller and more regular type Is boutons. Both types of boutons harbor multiple glutamatergic release sites that are spaced according to a strict nearest-neighborhood relationship (Atwood et al., 1993; Meinertzhagen et al., 1998; Sigrist et al., 2003) and that show discrete differences in their release probabilities (Kurdyak et al., 1994; Cooper et al., 1995). In spite of their close functional relationship, it has so far not been possible to systematically assess why both motor neurons maintain their obvious morphological and physiological differences and what their relevance is for the animal's behavior.

In order to shed light onto the poorly understood structure-function relationship of such discrete bouton-configurations, we used a combined theoretical and experimental approach to dissect the parameters that are biophysically important to support the natural function of discrete bouton configurations. We therefore developed and verified a three-dimensional model of synaptic bouton functions of larval NMJs of *Drosophila* and used it to systematically assess the meaning of experimentally rather inaccessible bouton parameters like bouton size, vesicle diffusion and vesicle release probability. We found that these parameters affect bouton output and its reliability of transmission on different time scales and that their individual combination in real boutons is predictive of the prevailing firing pattern that a given bouton type experiences.

## 2. Results

### 2.1. Morphological and physiological parameters to generate a model of glutamatergic boutons

**Figure 1:**
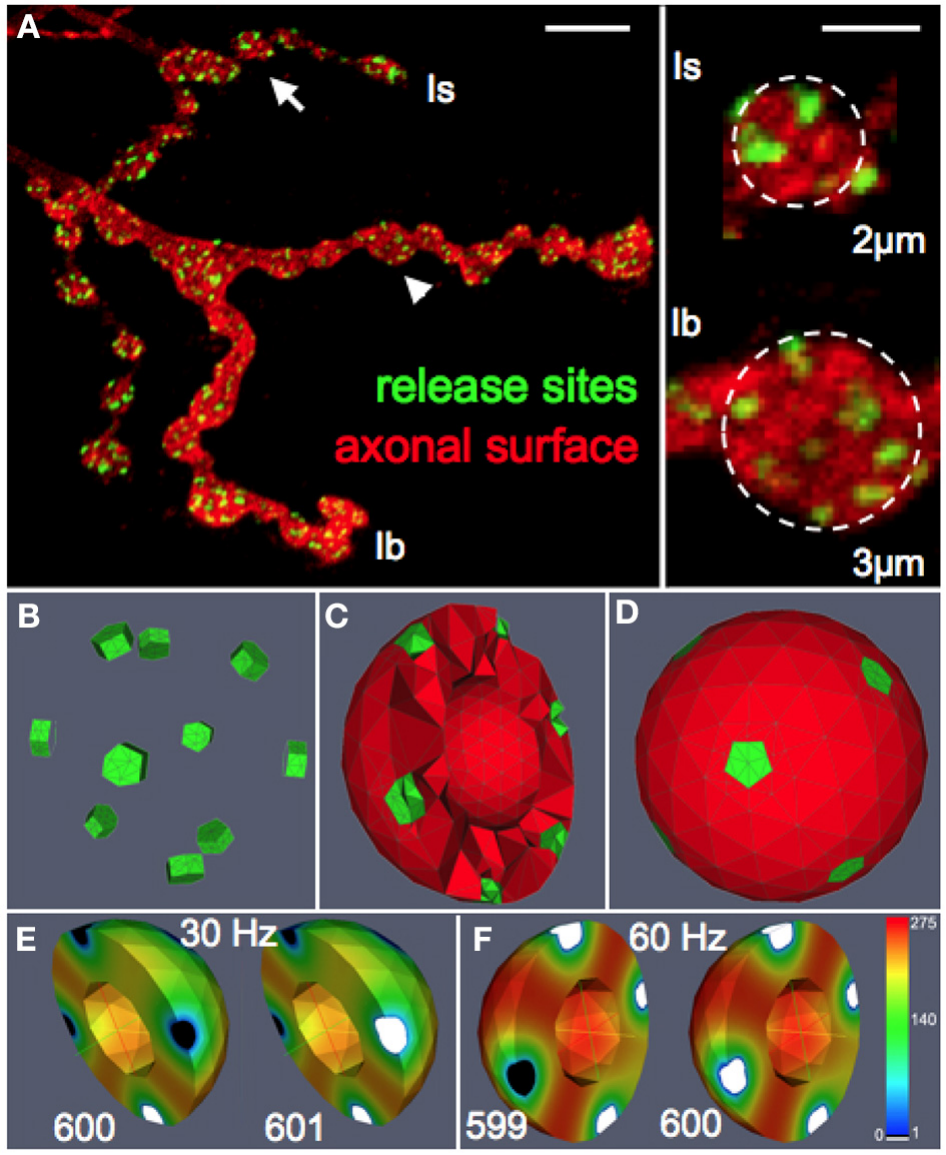
**Functional reconstruction of synaptic boutons of *Drosophila in silico*. (A)** Confocal image of a *Drosophila* larval neuromuscular junction (muscle 13) showing the synaptic terminals of two motor neurons that form type Is or Ib boutons, respectively. Release sites (green) were labeled with an antibody recognizing the active zone protein Bruchpilot (Wagh et al., 2006). The axonal surface (red) was labeled by anti-HRP immunoreactivity. Right panel: magnification of representative Is and Ib boutons (arrow and arrowhead) displaying an average diameter of 2 and 3 μm, respectively. Scale bars: 5 μm (left) 1.5 μm (right). **(B–D)** Release sites (green) and the vesicular diffusion area (red) are represented *in silico* by tetrahedral volume grids (Bastian et al., 2000). Release sites are positioned according to nearest neighborhood laws derived from ultrastructural data (Meinertzhagen et al., 1998; Sigrist et al., 2003). The bouton center is free of vesicles (Denker et al., 2009). **(E,F)** Simulation of vesicle dynamics in a Ib bouton (3 μm) during 30 Hz or 60 Hz stimulation (*P_o_* = 7%) with initial vesicle density of 275 μm^−3^. Encoded in color is the spatially resolved vesicle density (in vesicles/μm^3^) at the indicated stimulus number (599–601). Note that 60 Hz-stimulation rapidly depletes most release sites and their immediate surroundings of releasable vesicles (white) while more distant areas maintain high vesicle densities (red).

**Figure 2:**
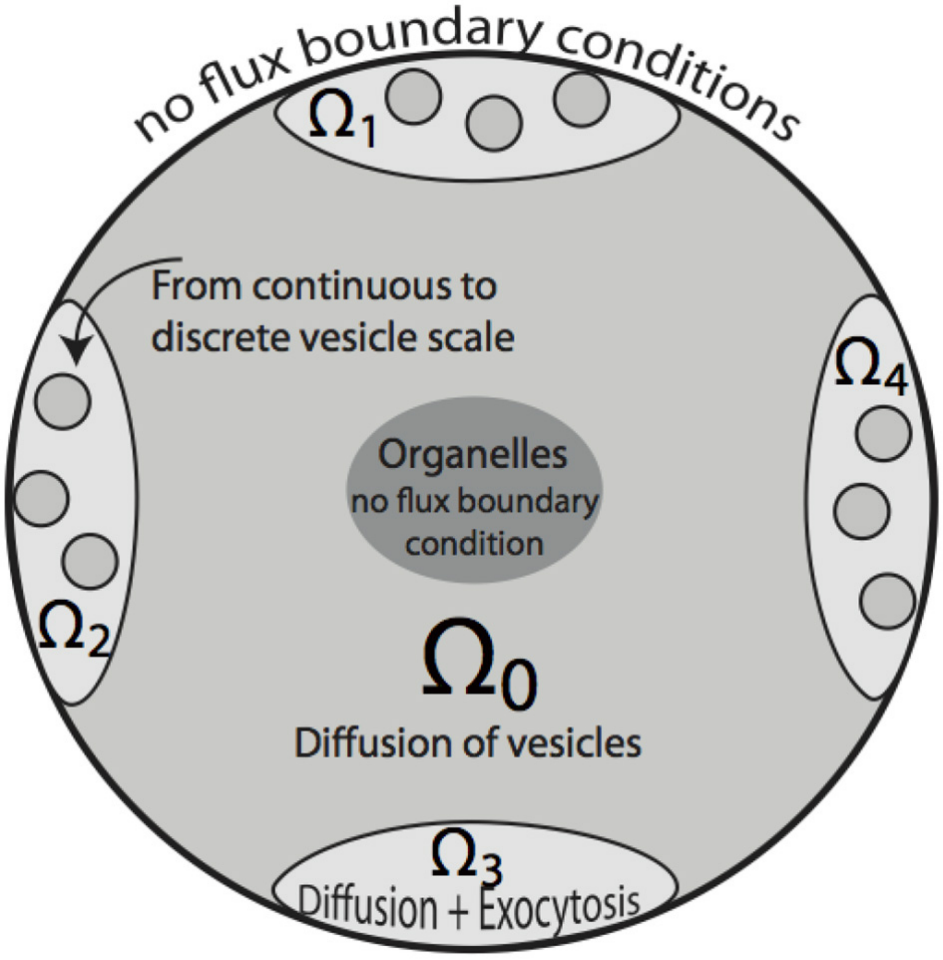
**Two-dimensional sketch of the bouton model**. Region Ω_0_ denotes the bouton area in which vesicle motion is governed by diffusion. A cutout region in the center represents space occupied by organelles, such as mitochondria. Ω_1_ … Ω_4_ define synaptic active zones. In these subdomains, vesicles diffuse and are subject to exocytosis modeled by the term *f*_syn_, that also includes the transition from the continuous to discrete scale in Ω_1_ … Ω_4_. The organelle as well as bouton boundaries are treated as no-flux boundary conditions.

To systematically assess the influence of morphological and physiological constraints on the performance of multi-release-site boutons we first established a functional reconstruction of such boutons *in silico* that aimed at simulating the vesicle dynamics within a bouton during trains of action potentials. From confocal image stacks of NMJs of third instar *Drosophila* larvae (Figure 1A) and representative measurements of bouton dimensions of type Is and type Ib synaptic terminals, we first estimated the typical bouton diameter of type Is and type Ib boutons to 2 and 3 μm, respectively (right panel in Figure 1A). In addition, some terminal boutons showed diameters of up to 5 μm. These different bouton morphologies were constructed in ProMesh (Reiter and Wittum, 2014), and discretized with a tetrahedral volume grid. The geometries represented spheres with a volume spared out at the center of each sphere representing organelle occupation, hence an area not occupied by vesicles (Figures 1C,D, 2). Each bouton geometry consisted of a zone Ω_0_ where vesicles were motile as well as exocytosis zones Ω_1_ … Ω_*n*_ where vesicles are motile and could be released (Figures 1B–D, 2). Taking into account the total number of vesicles that can be released from a NMJ during vesicle depletion experiments (Delgado et al., 2000) and data from ultrastructural analyses (Sigrist et al., 2002) we estimated a concentration of 150–500 vesicles per μm^3^ within a bouton at rest (see Materials and Methods for details). The positioning of the vesicle release sites on the surface of these spherical boutons was guided by serial ultrastructural reconstructions of larval boutons from which we determined the densities of T-bar harboring active zones between 0.37 and 0.46 μm^2^ (Meinertzhagen et al., 1998; Sigrist et al., 2002, 2003) with an average active zone diameter of 300–400 nm (Meinertzhagen et al., 1998; Sigrist et al., 2002, 2003). Based on these data we assumed 7 equally spaced active zones per Is bouton, 10 release sites per Ib bouton and up to 14 release sites in a large terminal bouton.

Synaptic active zones were represented by a multiedge-surface approximating a cylindrical structure with a diameter of 300–400 nm and a volumetric depth of 200 nm, which defines a volume that harbored roughly 6–8 vesicles, depending on the starting vesicle densities, which were chosen between 150 and 500 vesicles per μm^3^. An overview of the parameter ranges used in the simulations is given in Table 1. Where simulation data and results are shown, we specify which values within the given ranges were chosen. Within the active zones we made a transition from vesicle motion at the continuous scale to discrete vesicles that undergo exocytosis (see Materials and Methods for a detailed description). At this stage of the model we did not incorporate voltage-gated calcium channels on the plasma membrane and presynaptic calcium dynamics and its role in triggering vesicle fusion but instead modeled action potential triggered release of vesicles by the term *f*_syn_ that described a stochastic vesicle release process controlled by the stimulation frequency ω and a constant output probability *P_o_* throughout a given simulation. For literature-based simulations, we used *P_o_* = 29 and 7 % assigned to Is and Ib boutons respectively, values taken from Kurdyak et al. (1994); Cooper et al. (1995) instead of regulated *P_o_* values that are known to be involved in synaptic facilitation and depression. This restriction enabled us to dissect the role of each of these parameters on presynaptic vesicle dynamics under otherwise constant conditions. Although vesicle recycling has an effect on the transmission efficacy, we did not incorporate vesicle replenishment into the model in order to avoid introducing still unknown variables. Since the experiments also focus exclusively on preexisting vesicles, model and experiments are comparable.

**Table 1:** **Overview over the bouton configurations and parameter values used in this study**.

**Boutons**	**Is**	**Ib**	**Terminal bouton**
Bouton diameter	2 μm	3 μm	5 μm
Number of synapses	7	10	14
Cutout Radius	0.5 μm	0.8 μm	1.3 μm
**Values**	**Range**	**Stepwidth**	
*P_o_*	1–90%	1%	
ω	10–100 Hz	5 Hz	
Vesicle density	150–500 μm^−3^		

To implement into our model a meaningful simulation of vesicular motilities during trains of high-frequency action potential firing we made the following considerations: at *Drosophila* NMJs presynaptic vesicles have been assigned to three functionally distinct vesicle pools, the readily releasable pool, the recycling pool and the reserve pool of vesicles (Kuromi and Kidokoro, 2000; Denker et al., 2009). Although the number of vesicles belonging to each vesicle pool and their respective motilities are very different at rest (Kuromi and Kidokoro, 2000), stimulation frequencies above 10 Hz have been shown to be sufficient to recruit vesicles from all pools for release (Kuromi and Kidokoro, 2000; Wagh et al., 2006; Denker et al., 2009). Since the natural firing pattern of larval motor neurons uses typically frequencies higher than 10 Hz (**Figure 6**) all vesicles within a bouton have to be considered mobile during natural activity patterns *in vivo*. It is, however, currently not clear whether vesicle motility during high frequency stimulation follows a simple diffusion process (Gaffield et al., 2006) or whether it underlies active transport mechanisms requiring cytoskeletal elements (Ryan, 1999; Jordan et al., 2005; Tokuoka and Goda, 2006; Seabrooke et al., 2010; Kisiel et al., 2011). We have previously shown that the presynaptic actin-based cytoskeleton within *Drosophila* boutons is highly dynamic with major rearrangements occurring on a rapid time scale (Steinert et al., 2006). These cytoskeletal rearrangements may result in a constant intermixing of presynaptic vesicles and hence in a dynamic and overall rather homogeneous distribution of vesicles within the presynaptic terminal. Based on these and the above observations we first assumed that all presynaptic vesicles are mobile during stimulation frequencies higher than 10 Hz (Kuromi and Kidokoro, 2000). We, secondly, assumed that during such stimulation periods vesicle motility may be approximated by undirected diffusion-like movements (Shtrahman et al., 2005; Darcy et al., 2006; Fernandez-Alfonso and Ryan, 2008; Westphal et al., 2008). We therefore used an effective diffusion coefficient of *D* = 5 · 10^−3^ μm^2^/s (Shtrahman et al., 2005) that included stick-and-diffuse processes that have been shown to occur during interactions of vesicles with the cytoskelleton (Darcy et al., 2006; Fernandez-Alfonso and Ryan, 2008; Westphal et al., 2008).

The above considerations formed the basis for the formulation of the diffusion equation and the sink term that were solved on complex morphologies in three-dimensional space using numerical discretization methods and solvers (see Materials and Methods section for details). All numerical methods were part of the simulation platform uG (Bastian et al., 2000). Numerical simulations of our model generated three-dimensional density profiles of presynaptic vesicles that allowed us to monitor the local distribution and dynamics of mature vesicles as a function of bouton diameter, the applied stimulation pattern and the vesicle release probability (Figures 1E,F, Supplemental Video S1).

### 2.2. Disproportional depletion of vesicle release at high stimulation frequencies is due to limited vesicle diffusion

**Figure 3:**
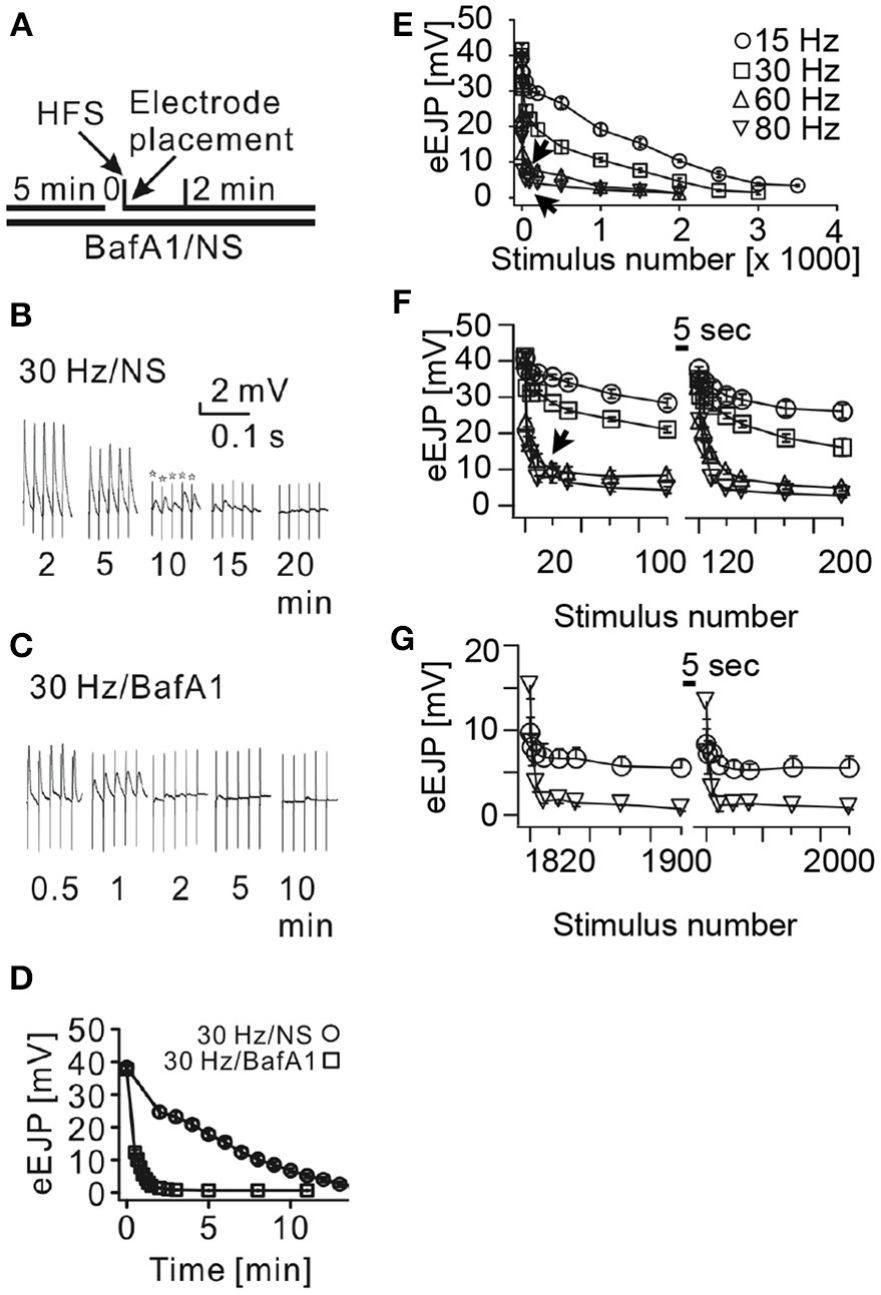
**Disproportional depletion of eEJPs is due to limited availability of presynaptic vesicles**. **(A)** Experimental design: larval filet preparations were pre-incubated in normal saline (NS) or NS containing 2 μM Bafilomycin A1 (BafA1), a blocker of the vesicular proton pump, to inhibit transmitter filling in recycling vesicles. Motor nerves were then continuously stimulated with a suction electrode at various frequencies (HFS) while we simultaneously recorded the membrane potential of the postsynaptic muscle fiber 6. **(B,C)** Representative traces of these recordings in the absence or presence of BafA1 taken at the indicated time-points of stimulation (stars: stimulation artifacts). **(D)** Quantification of eEJP-depletion dynamics during continuous 30 Hz stimulation with and without BafA1 incubation. Note, that eEJPs could be detected over at least 15 min of 30 Hz-stimulation in controls without BafA1-treatment (**B** and circles) whereas eEJPs of BafA1-treated preparations depleted within 2 min (**C** and squares). **(E–G)** eEJP-depletion dynamics in BafA1-treated preparations caused by the indicated stimulation frequencies and expressed as a function of the stimulus number. **(E)** Shows the eEJP-depletion dynamics during continuous long-term stimulation. In **(F,G)** stimulation trains of 100 stimuli were separated by pausing intervals of 5 s. Note that the depletion of eEJP-amplitudes was disproportionally faster during 60 and 80 Hz stimulations compared to 15 and 30 Hz stimulations. Short pausing intervals resulted in an almost complete recovery of eEJP-amplitudes at the beginning of the experiment **(F)**. In later phases of the experiment **(G)** the eEJP recovery was significantly higher in 80 Hz stimulated NMJs than in 15 Hz stimulated NMJs (*p* < 0.033 at stimulus 1800 and *p*<0.019 at stimulus 1900). Data in **(D–G)** represent means ± SEM of 4 to 6 independent preparations.

To verify the validity of the parameters of our bouton model we wished to compare its behavior in a task that is similar to a simple *in vivo* experiment. We therefore chose an experimental vesicle depletion paradigm that is triggered by continuous high-frequency stimulation of larval motor nerves in the presence of a blocker of the vesicular proton pump, Bafilomycin A1 (Kuromi and Kidokoro, 2000) (BafA1 in Figure 3A). Synapses in BafA1-treated larval filet preparations fail to fill recycling presynaptic vesicles with the neurotransmitter glutamate and hence can be used in intracellular recording experiments of postsynaptic membrane potentials to exclusively visualize the release of preexisting mature vesicles. As expected, the amplitudes of evoked excitatory junctional potentials (eEJPs) of BafA1-treated larval preparations depleted significantly faster during continuous 30 Hz nerve stimulation than in untreated preparations (Figures 3B–D). The here observed rate of eEJP-deletion in BafA1-treated preparations was similar to the rate of vesicle depletion in temperature-sensitive mutants of dynamin (shibire^ts^) (Siddiqi and Benzer, 1976) that at restrictive temperatures inhibit all endocytic processes (Delgado et al., 2000) indicating that in spite of the different molecular mechanisms of both treatments the measurable outcome was comparable. In addition, we observed that the eEJP-depletion progressed at rather slow rates during stimulation frequencies between 15 and 30 Hz, whereas at higher stimulation frequencies (60–80 Hz) the initial depletion was disproportionately faster (arrows in Figure 3E). This unexpected phenomenon was unlikely due to desensitization of postsynaptic glutamate receptors, since the quantal amplitudes (miniature excitatory junctional potentials, mEJPs) were unaltered under all tested conditions (data not shown). Instead, the latter observation suggested that a presynaptic limitation was responsible for the disproportionally rapid decay of eEJP amplitudes at high stimulation frequencies.

An analysis of NMJs that transmitted naturally produced motor patterns in the absence of BafA1 (Supplemental Figures S1A,D) revealed that these synapses were capable of maintaining their eEJP amplitudes throughout entire patterns (Supplemental Figures S1C,F) even when the firing frequencies were in the range of 60–80 Hz (Supplemental Figures S1B,E). This result demonstrated that high-frequency firing patterns could be faithfully transmitted by the glutamatergic boutons of larval NMJs. It further suggested that the disproportionally rapid decay of eEJP amplitudes seen in BafA1-treated synapses might be due to limited availability of preexisting mature vesicles at active zones. If such a limited availability of transmitter filled vesicles would cause the disproportional synaptic depression during 60–80 Hz stimulation rates one would expect that short pausing intervals between stimulation trains allow distant mature vesicles to functionally reoccupy release sites over time resulting in recovered eEJP amplitudes. If BafA1-treatment would potentially unspecifically block other synaptically relevant processes such as calcium export or cytoskeletal organization that would disproportionally stronger affect high frequency transmission, eEJP amplitudes should continue to show depression after a paused stimulation. This assumption is contradicted by our results in Figures 3F,G showing transient depression at all stimulation frequencies excluding a persistent pleiotropic defect induced by the procedure. In experiments in which trains of 100 stimuli were separated by pausing intervals of 5 s we found an almost complete recovery of the eEJP amplitudes at all used stimulation frequencies (15–80 Hz) immediately after the pause (Figure 3F). Furthermore, the longer this mode of stimulation continued the more the recovered eEJP amplitudes deviated between high and low frequencies (Figure 3G): eEJP recovery was significantly larger in boutons after 1800 stimuli at 80 Hz than at 15 Hz (*p* < 0.033, *n* = 4) or after 1900 stimuli (*p* < 0.019, *n* = 4). Both observations were consistent with the idea that vesicle motility was limiting the availability of mature vesicles at active zones. This leads during high frequency stimulation to a rapid failure to reoccupy empty active zones with mature vesicles and thus to a rather small total number of released vesicles per train. The higher number of remaining mature vesicles is then responsible for the strong eEJP recovery even after prolonged stimulation periods (triangle in Figure 3G). Conversely, during lower stimulation frequencies vesicle release and active zone reoccupation appear to be more balanced resulting in a higher total vesicle output per train and consequently in a weaker eEJP recovery after prolonged stimulation (circle in Figure 3G).

#### 2.2.1. Verification of the model

**Figure 4:**
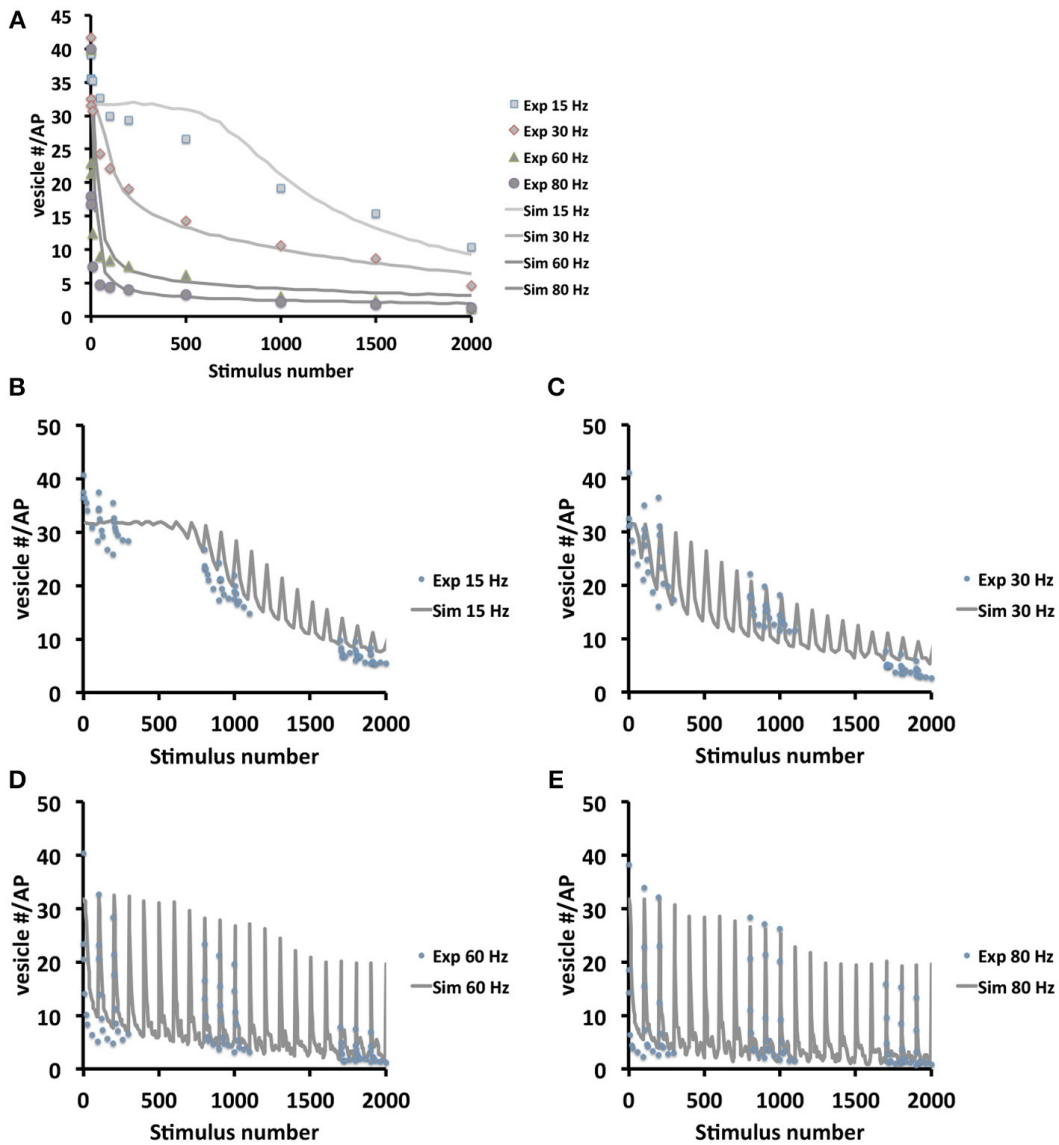
**Model validation at different frequencies and stimulation protocols**. **(A)** Comparison between experimental and simulated data at the frequencies 15, 30, 60, and 80 Hz. In order to compare simulation and experiment, the initial vesicle density was computed from the experimentally recorded vesicle release for each frequency and thus initial vesicle densities were set to 500, 280, 190, and 150 vesicles/μm^3^ respectively in the simulations. Note that in the beginning 1000 stimuli at 15 Hz the depletion is considerably faster in real boutons than in the simulation. This may indicate that 15 Hz stimulation is initially not sufficient to efficiently mobilize all presynaptic vesicles e.g., from the reserve pool of vesicles. This limitation is overcome at higher stimulation frequencies. **(B–E)** Experimental (dotted) and simulation (lines) data in stop and go stimulation protocol, with 100 stimuli and 5 s pause, show decline in in the postsynaptic amplitudes (**B,C**: simulation data averaged over 25 stimuli; **D,E**: simulation data averaged over 5 stimuli). The plots show that with increasing frequency (from **B–E**) amplitudes decrease slower, due to higher exocytosis failure rates at high frequencies caused by diffusion limited active zone replenishment. Note that similar to **(A)** the experimental depletion is faster than the *in silico* depletion. As above this may be due to a not efficient mobilization of presynaptic vesicles at 15 Hz.

To further verify the quality of our model we systematically compared simulated and experimental vesicle depletion curves of different conditions (Figure 4). For the model we used as before parameter values from previous experimental studies (Kurdyak et al., 1994) and set up our model with 6 Ib and 13 Is boutons with *P_o_* values of 7 and 29% for the release sites of type Ib and type Is boutons, respectively (Kurdyak et al., 1994). Under these conditions the *in silico* simulations of the vesicle depletion experiments approximated the experimental observations in that the simulated total amount of vesicular release decreased at various stimulation frequencies with similar time courses as in the *in vivo* experiments (Figure 4). Intriguingly, we identified the same two features that together could explain the disproportionally faster depletion of vesicle release at high stimulation frequencies with our model simulation of the local vesicle dynamics within a bouton (Figures 1E,F): first, 600 stimuli applied at 60 Hz depleted most release sites and their immediate surroundings of releasable vesicles (white patches in Figure 1F) whereas the same number of 30 Hz-stimuli left more release sites functional with one or more releasable vesicles (Figure 1E). Second, 600 stimuli at 60 Hz caused a decrease of the overall number of vesicles per bouton to about 84% (mostly red areas in bouton lumen of Figure 1F), whereas the same number of stimuli applied at 30 Hz resulted in a stronger overall decrease to about 73% (mostly yellow in Figure 1E). Since we modeled vesicle dynamics during synaptic stimulation as an undirected diffusion process that matched our experimental observations remarkably well, it seemed likely that vesicle diffusion is an important limiting factor during high frequency stimulation episodes *in vivo* (see Discussion). This included the disproportionally faster depletion of vesicle release at 60 or 80 Hz compared to lower frequencies (Figure 4A). We further compared experimental and simulation data in stop and go experiments (from Figures 3F,G), where we applied 100 stimuli at the given frequency and 5 s stimulation pause alternatingly (see also Figures 3F,G). Data shown in Figures 4B–E showed that the peak amplitudes decreased with increasing stimulus number, as expected for all stimulation frequencies. We found that both experiment and simulation showed a rapid recovery from depletion during the pausing periods (Figures 4B–E). In both cases the recovery was considerably larger during high-frequency stimulation (Figures 4D,E) than at lower frequencies (Figures 4B,C). This could be attributed to the fact that the overall failure rate in vesicle release (i.e., a release event is triggered, but no vesicle is present at the active zone) was larger at high frequencies, which in response causes a slower decline in postsynaptic response. This effect was a further indicator for vesicle diffusion limiting synaptic response.

In order to quantify the results in Figure 4A, we applied different measures (least-squares and *L*_2_-norm, see Materials and Methods for more detail) to the experimental and simulated data (Table 2). These similarities demonstrate that our model includes the necessary biophysical laws that explained the stimulation-induced vesicular dynamics of multi-release-site boutons in space and time and hence might be used to analyze vesicle movement and their potential restriction for release at high temporal and spatial resolution. We saw that the overall bouton dynamics *in vivo* and *in silico* show significant similarities, though the diffusion-based *in silico* results demonstrated a slower decay phase at high frequency stimulation in the initial stimulation phase, thus indicating that pure diffusion might not suffice for high amplitude/high frequency output (see Discussion).

**Table 2:** **Comparing the experimental and simulated data at different frequencies and with the least-squares method *S* and *L*_2_-norm (see Materials and Methods) shows good agreement between experimental and simulation data shown in Figure 4A**.

**Error norm/frequency**	**15 Hz**	**30 Hz**	**60 Hz**	**80 Hz**
Least-squares *S*	0.03	0.02	0.07	0.08
*L*_2_-norm	0.11	0.04	0.15	0.1

#### 2.2.2. Bouton performance is differentially affected by bouton size, release probability, and stimulation frequency

**Figure 5:**
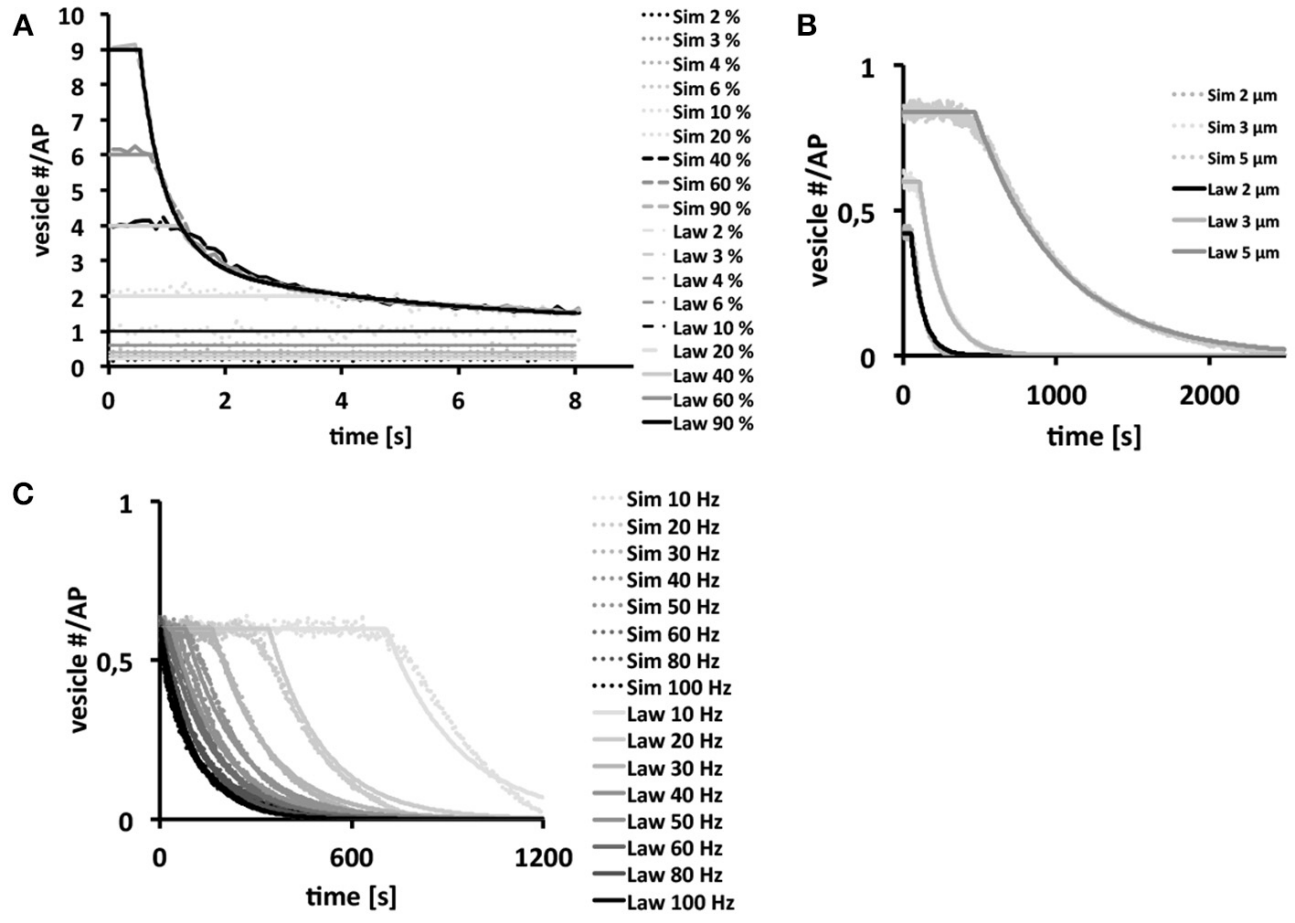
**Bouton output and output endurance are differentially affected by stimulation frequency, bouton size, and *P_o_***. Vesicle density is set to 400 μm^−3^. In each figure row only one of these three parameters were varied, while keeping the other two fixed. Systematic analysis of the effects of changes in *P_o_* **(A)**, bouton diameter (*d* = 2, 3 and 5 μm) with 7, 10, and 14 release sites respectively **(B)** and stimulation frequency **(C)** on the simulated total output dynamics over several simulations (dotted) or on derived biophysical depletion laws (lines). If not indicated otherwise we used a *P_o_* of 6%, a bouton diameter of 3 μm and a stimulation frequency of 40 Hz. Note that the total bouton output is initially very large at high *P_o_*-values, but it rapidly depletes to the output of low-*P_o_* boutons that continue reliable transmission. Further note that larger boutons harbor more release sites resulting in larger and longer-lasting total outputs **(B)**. Stimulation frequencies up to 40 Hz can be transmitted reliably over some time (plateaus in **C**) while higher frequencies can only be maintained for short time periods. Interestingly, the long term behavior is independent on the choice of *P_o_* for *P_o_* ≥ 5 %.

Since our three-dimensional model described the vesicle dynamics in a multi-release-site bouton during example stimulation frequencies similar to the experimental results, we systematically varied each individual component using as standard settings *P_o_* = 6%, *d* = 3 μm (10 active zones) and ω = 40 Hz, with initial vesicle density set to 400 μm^−3^, in order to dissect their respective roles in determining the functional properties of different bouton types [Figure 5 (dotted)]. From these simulations we derived empirical laws [Figure 5 (lines)] as a heuristic fit of the *in silico* data (see Materials and Methods) in order to assist the interpretation of our simulations.

We found that high *P_o_*-values strongly affected the magnitude of the overall vesicle release at the beginning of a 40 Hz stimulation train (Figure 5A). However, the strong initial vesicle release disappeared within a second of stimulation due to the rapid depletion of vesicles at individual release sites. Thus, high *P_o_*'s seemed only meaningful if large amplitude outputs were needed for short time periods, whereas small *P_o_*'s provided long-term reliability of smaller amplitude outputs (Figure 5A). The size of boutons affected its performance under otherwise constant conditions (*P_o_* = 6%; ω = 40 Hz) on a rather long time-scale. Bigger boutons with correspondingly more release sites showed larger outputs that depleted significantly slower than those of smaller boutons (Figure 5B). Thus, larger boutons seemed to be better suited for sustained periods of synaptic activity whereas smaller boutons with fewer release sites were only reliable during short trains. Note that the term “reliable transmission” refers to the phases in which vesicle exocytosis is constant, thus the phase in which the system is not limited by high stimulation frequencies, bouton size or vesicle output probability.

Our analysis of the effects of a broad range of stimulation frequencies revealed that frequencies of up to 40–50 Hz were reliably transmitted by our standard type Ib bouton for seconds (initial plateaus in Figure 5C). At higher stimulation frequencies reliable vesicle release was limited to only few stimuli until it becomes diffusion-limited (see Figure 5C). Thus, type Ib boutons could transmit a broad range of stimulus frequencies even in the absence of vesicle recycling, however, frequencies above ≈50 Hz could only be maintained for very short time-periods (Figure 5C).

The data in Figure 5B, with initial vesicle density set to 400 μm^−3^ and 7 active zones for the Is bouton, show that the exocytosis dynamics are separated into a stable phase in which exocytosis is constant and an unstable phase in which there is a decay in vesicle exocytosis. The law in Figure 5C shows that in the range above 50 Hz the system becomes unstable nearly instantaneously. Experimentally this effect is observed at around 45 Hz for an output probability estimated at roughly 7%.

#### 2.2.3. Decomposition of the compound response of Is/Ib-harboring NMJs

**Figure 6:**
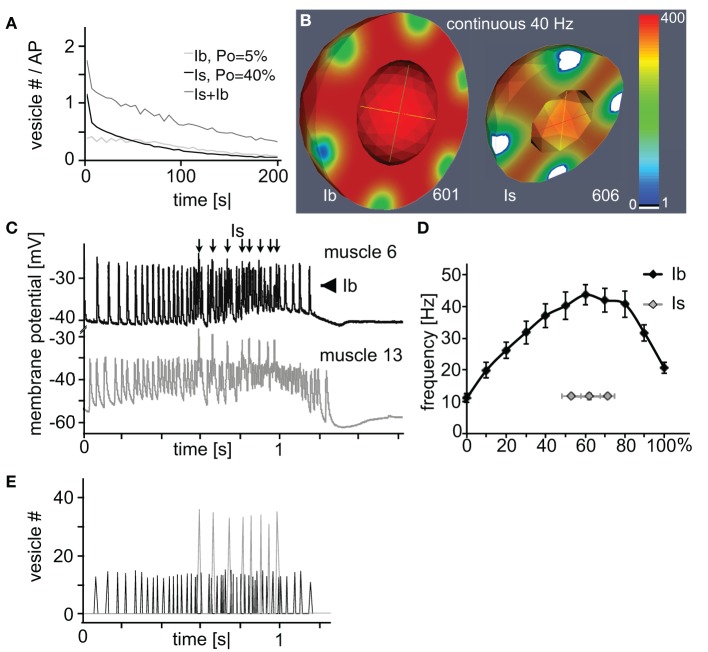
**Rational design of a naturally performing NMJ *in silico***. **(A,B)** Simulation of the total **(A)** or spatially resolved **(B)** vesicle dynamics of a type Ib (3 μm, 10 active zones; *P_o_* = 5%) and Is (2 μm, 7 active zones; *P_o_* = 40%) bouton during continuous 40 Hz-stimulation. In both cases the initial vesicle density was set to 400 μm^−3^. **(A)** Under these conditions the total output dynamics of combined Is and Ib boutons showed a characteristic biphasic depletion. **(B)** Is boutons showed more depleted release sites than Ib boutons at similar stimulus numbers. **(C)** Simultaneous recordings of the natural larval motor pattern in muscles 6 and 13 identified a synchronous firing pattern (arrows) originating from one motor neuron forming Is boutons on both muscles. The remaining asynchronous firing (arrowheads) originates from muscle specific motor neurons forming Ib boutons. **(D)** Dissected frequency profile of natural larval motor patterns plotted as a function of the relative train length. Data represent means ± SEM from 10 identified Is- and 10 Ib-motor patterns from muscle 6. **(E)** Simulation of the vesicle output of an *in silico* NMJ (17 3 μm boutons, *P_o_* = 7%; 19 2 μm boutons (7 active zones), *P_o_* = 29%). Initial vesicle density was set to 275 μm^−3^. This NMJ exocytosis pattern was driven by the brain governed motor pattern as measured experimentally and presented in **(D)**.

Since all three analyzed parameters affected bouton performance in distinct manners it was tempting to combine these individual model-derived characteristics into realistic functional profiles of the two larval bouton types. If one assumed that the active zones of the smaller type Is boutons operated with a rather high vesicle release probability (in the range of *P_o_* = 40%) while the bigger type Ib boutons used a lower, in the range of *P_o_* = 6%, (Kurdyak et al., 1994; Lnenicka and Keshishian, 2000) our above simulations allowed clear predictions as to how the output of a NMJ with roughly similar numbers of both bouton types will look like (Figure 6A): a continuous stimulation of, e.g., 40 Hz with initial vesicle density set to 400 μm^−3^ will result in a compound junctional output (dark gray line) that is initially dominated by the strong release from Is boutons (black line) before their contribution decays due to rapid vesicle depletion at their active zones (white patches in Figure 6B). In contrast, the type Ib boutons of this NMJ are expected to show reliable but small amplitude performance (gray line in Figure 6A) with no extensive signs of diffusion-based vesicle depletion (left bouton in Figure 6B).

Intriguingly, in our *in vivo* experiments in which we stimulated the axons of both motor neurons synchronously at various frequencies (Figure 3E) we observed a very similar behavior of the compound response as indicated by an initial steep decrease in the eEJP amplitudes followed by an abrupt transition (arrows in Figures 3E–G) into a slower decay phase. These results suggested both *in silico* and *in vivo* that type Is boutons were not well suited to reliably transmit sustained high-frequency stimuli and they indicate that it was unlikely that in a behaving animal the natural motor pattern fires type-Is and -Ib boutons synchronously.

#### 2.2.4. The natural firing pattern matches the functional properties of type Is and Ib boutons

**Figure 7:**
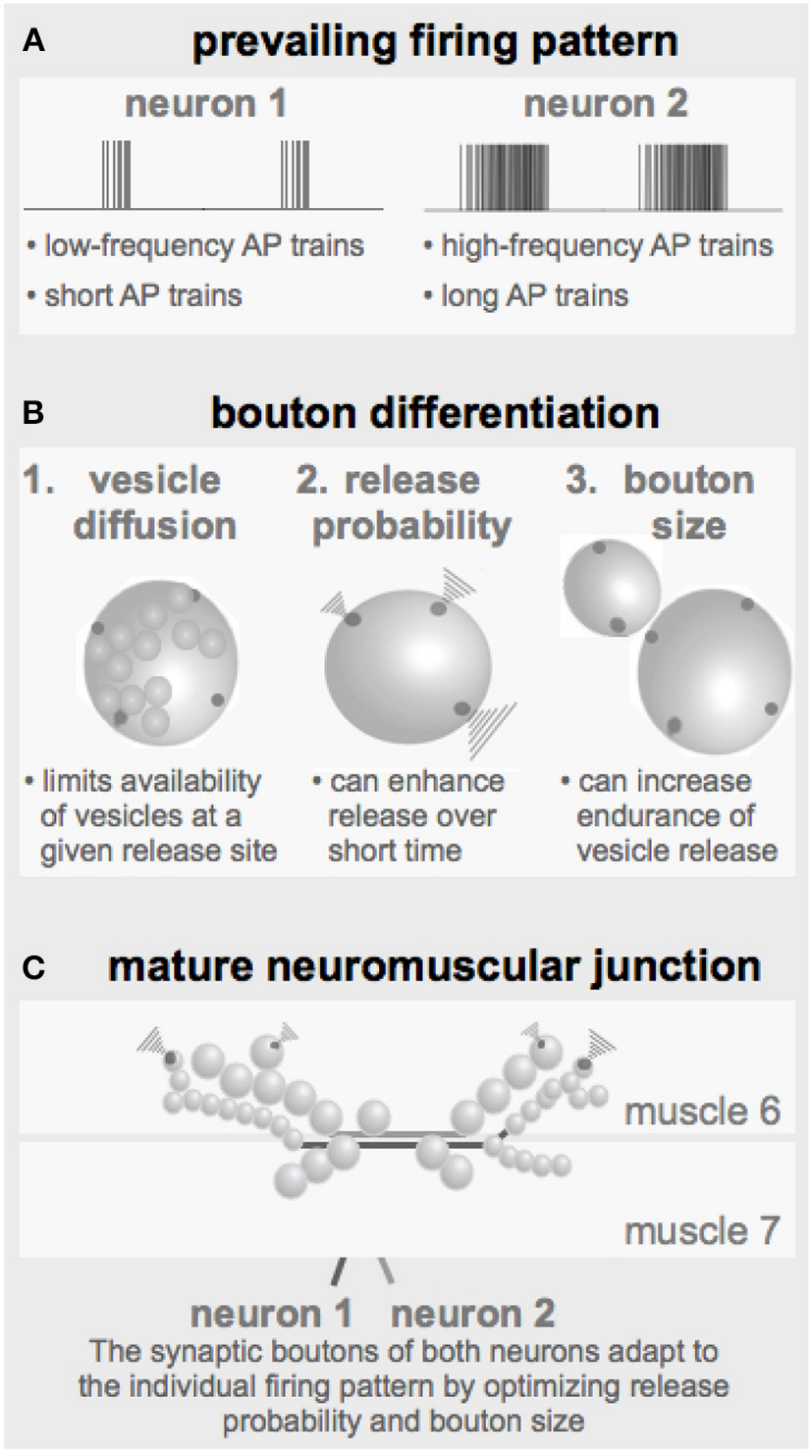
**Parameters defining the configuration of a NMJ**. **(A)** The prevailing firing pattern of a neuron sets the standard for the functional properties of its synaptic boutons. **(B)** In order to guarantee reliable transmission of a given firing pattern the already existing synaptic boutons may modify their genetically determined layout by adapting the here identified critical parameters such as the release probability and the bouton size (together with the number of release sites). **(C)** Throughout larval development individual boutons are added to the terminals of both motor neurons resulting in strings of boutons with typical morphologies.

Our above results predicted that Ib boutons are likely utilized to transmit longer-lasting high-frequency stimuli whereas Is boutons are better suited for few low-frequency events. We tested these predictions by analyzing the natural firing pattern that is generated by the larval central pattern generator to trigger coordinated muscle contraction. In order to differentiate between the patterns transmitted by Is and Ib boutons we established simultaneous recordings from the neighboring muscles 6 and 13 that shared a common Is-innervation from the same motor neuron but received an independent Ib-innervation each from two distinct motor neurons (Lnenicka and Keshishian, 2000). We found that the natural motor pattern consists of two discrete components (Figure 6C): a tonic eEJP pattern originating from type-Ib boutons (arrowheads) that displayed in each train a bell-shaped frequency ramp of pulses ranging between 10 and 45 Hz on muscle 6 (black line in Figure 6D; 50.8 ± 5.4 pulses per train, mean ± s.e.m. of *n* = 10). The second component originated from type-Is boutons and consists of only few large pulses at a rather stable frequency of about 12 Hz (arrows in Figure 6C; gray line in Figure 6D; 5 ± 0, 7 pulses per train, *n* = 10). These results demonstrated that Is or Ib motor neurons fire with patterns that corresponded well to our above predictions of their individual physiological properties (see Discussion and Figure 7). Conversely, when we fed the naturally observed firing pattern of both boutons into our model we found that the *in silico* boutons reproduced the respective patterns consistently even in the absence of vesicle recycling (Figure 6E).

## 3. Materials and methods

### 3.1. Electrophysiological recordings

Electrophysiological recordings of membrane potentials of body wall muscle 6 were performed on filet preparations of size-matched mid third instar male larvae at 22C. Dissected larvae were first immersed in normal saline (NS) solution containing 2 mM Ca^2+^ (Jan and Jan, 1976) that was then replaced by NS containing 2 μM Bafilomycin A1 (BafA1). After 5 min incubation muscle 6 of segment A_2−3_ was impaled with a 1530 MΩ microelectrode filled with 3 M KCl. For stimulation the cut end of the intersegmental nerve of this segment was placed into a suction electrode and suprathreshold stimuli were applied at the indicated frequencies with or without intermitting pausing intervals. Cells with a resting potential more negative than −60 mV and an input resistance R_*in*_ of 5 MΩ were used for further analysis. Data analysis was performed off-line (Clampfit10, Axon Instruments). Recordings of natural motor patterns were performed similarly (NS containing 2 mM Ca^2+^ without BafA1) except that the motor nerves were left attached to the ventral nerve cord and that we recorded simultaneously from muscle 6 and muscle 12 or 13. This configuration allowed us to differentiate the firing patterns of type 1b- and 1 s-innervations (Chouhan et al., 2010). Only those trains were taken for further analysis that correlated with a peristaltic contraction wave of the segmentally repeated body wall muscles. eEJP-amplitudes in Supplemental Figure S1 were measured from the extrapolated resting potential to the peak of the eEJP.

### 3.2. Parameters used in the model

#### 3.2.1. Bouton size

Paraformaldehyde fixed larval filet preparations were immuno-labeled with antibodies recognizing a neuronal surface epitope (anti-HRP) and the active zone protein Bruchpilot (Wagh et al., 2006). Confocal images of 12 of such labeled M12/13-NMJs (e.g., Figure 1A) were used to estimate the diameters of synaptic boutons of type Is motor neurons and type Ib motor neurons using the measurement tool of ImageJ. As exemplified in Figure 1A bouton sizes varied more in type Ib branches than in type Is branches but they were on average larger than type Is bouton. Terminal boutons showed particularly large diameters. Because of these considerable variances we chose to confine our current analysis to three representative classes of bouton sizes with the following diameters: Is: 2 μm; Ib: 3 μm; terminal bouton: 5 μm.

#### 3.2.2. Vesicle density

The vesicle densities used in our model were determined as follows: (1) The total number of quanta that can be released from a Drosophila NMJ has been previously determined to be in the range of 85,000 (Delgado et al., 2000). This translates to 850–1700 vesicles per bouton for NMJs with 50–100 boutons and a concentration of 130–270 vesicles/μm^3^ for an average bouton (diameter: 2.5 μm, inner vesicle free diameter: 1.5 μm). (2) Ultrathin-sectioned larval boutons showed on average 1–2 vesicular profiles every 200 nm (Sigrist et al., 2000; Steinert et al., 2006) mounting up to about 500–1000 vesicles/μm^3^. For our simulation experiments we have chosen a vesicle concentration between these estimates: 150–500 vesicles/μm^3^.

#### 3.2.3. Output probability *P_o_*

The output probability *P_o_* values used in this study correspond to the experimentally determined vesicle release probabilities *P* of synapses of the two bouton classes Is and Ib that were estimated to average *P* values of 29 and 7%, respectively (Kurdyak et al., 1994; Cooper et al., 1995). Although a large body of evidence suggests that vesicle release probabilities are non-uniform and subject to change during facilitation and depression phenomena we chose in this stage of our model to simplify and keep the *P_o_* values constant during each simulation. To still account for such changes we assayed the effects of different *P_o_* values in a bandwidth of 1–90%. The *P_o_* in our model describes the overall likelihood that a given vesicle is released from a given active zone and therefore represents a summed value for all processes involved in an individual vesicle release event including vesicle docking and priming, the functional status of voltage gated calcium channels, presynaptic calcium dynamics and calcium triggered vesicle fusion.

### 3.3. Diffusion-reaction model for vesicle dynamics

The mathematical model presented in this manuscript is based on the coarse-scale model of brownian particle motion proposed in Einstein (1905). Using this approach we can deduce that vesicle dynamics can be described by means of a diffusion-reaction equation. To achieve this result, a fixed control region *V* ⊂ ℝ^3^ and an interval of time 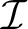 = [0, *T*] are considered. The total number of vesicles contained in *V* at time *t* ∈ 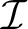 is calculated as

(1) $$N_{V}(t)=\int_{V}c(x,t)\text{d}v,$$

where *c* (units [*c*] = vesicles · μm^−3^) is the concentration of the vesicles. The variation of *N*_*V*_ over time is regulated in principle by the flux of vesicles through the boundary ∂*V*, a source and a sink term, i.e.,

(2) $${\text{d}}_{t}N_{V}=-\int_{\partial V}J\cdot n\text{d}\sigma +\int_{V}\left(R-A\right)\text{d}v.$$

Assuming regularity, we can extend *J* to the interior of the control volume *V*. Using Gauss' Theorem, and a standard localization argument, yields the local form of (2):

(3) $$\partial_{t}c=-\nabla \cdot J+R-A.$$

In (2) and (3), *J* represents the vesicle flux vector, while *R* and *A* represent, respectively, recycling and absorption of vesicles. In general, both *R* and *A* depend on concentration and may also exhibit explicit dependence on time and spatial coordinates. Since *A* should vanish when concentration is zero, it is given through an expression of the type *A*(*c, x, t*) ≥ 0, such that the condition *A*(0, *x, t*) = 0 applies. The physical units of *A* are [*A*] = vesicles/(μm^3^ s). For the purposes of our manuscript, recycling is switched off (i.e., *R* is set equal to zero from the outset), and only the depletion of vesicles is accounted for. Using Fick's Law, and assuming isotropic diffusion, the vesicle flux vector can be expressed by *J* = − *D*∇*c*, with *D* the diffusion coefficient, and Equation (3) can be rewritten as

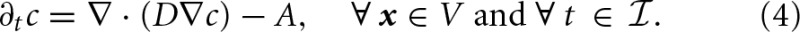



The computational domain, over which (4) is solved, is denoted by Ω ⊂ ℝ^3^ and coincides with the region of space occupied by a bouton. The set Ω is partitioned in subdomains Ω_*i*_, *i* = 0, …, *S*, with *S* being the number of synapses. Ω_0_ is the subdomain containing vesicles which are not part of synaptic regions and accounts for the largest part of the bouton. For *i* = 1, 2, … *S*, the Ω_*i*_ are the synaptic regions. The computational domain Ω therefore is written as

(5) $$\Omega =\Omega_{0}\cup \left(\overset{S}{\underset{i\;=\;1}{\cup }}\Omega_{i}\right),$$

see Figure 2. For all outer boundary surfaces, we use constant Neumann zero boundary conditions:

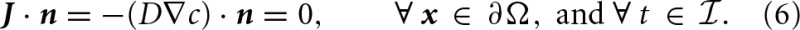



Finally, we close the mathematical problem by prescribing the initial condition

(7) $$c(x,0)=c_{0},\;\forall \;x\in \Omega ,$$

where *t* = 0 denotes the origin of time.

### 3.4. The sink term

The reason we use a sink term rather than a Neumann flux boundary condition to simulate vesicle exocytosis is that such a boundary condition would describe the situation in which the vesicles are permitted to cross the membrane of the bouton. However, this is not the observed mechanism of vesicle depletion in a bouton. Indeed, when the signal of the action potential reaches a synaptic region, the vesicles available for exocytosis fuse with the inner surface of the bouton and release neurotransmitters, which, in turn, are allowed to cross the membrane of the bouton. Therefore, in spite of a zero Neumann boundary condition for the vesicle flux, the total number of vesicles contained in a bouton is not conserved over time. To account for the fusion of the vesicles with the membrane of the bouton, we introduce a sink term *f_syn_* ≡ *A* that, under the conditions discussed below, removes one or no vesicle per action potential from each synaptic region of the bouton.

Vesicle exocytosis is a discrete event that reduces the pool of vesicles by an integer number of exocytosed vesicles. The sink term defined in the following models exocytosis based on the general synaptic vesicle release probability *P_o_* and a stochastic probability *P*_*i*_ that is calculated for each synapse in each simulation time step. Since vesicle motion is modeled on the continuous scale, and vesicle exocytosis relates to single vesicles, the sink term needs to couple the continuous scale with the single particle scale. This leads us to the following definition of the vesicle releasing sink term *f_syn_*:

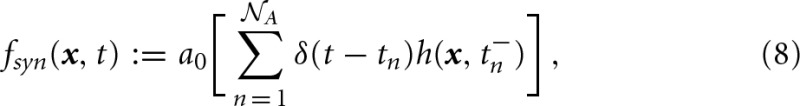



(9) $$\begin{array}{l} h(x,t_{n}^{-}):=\sum_{i\;=\;1}^{S}\Theta \left(4^{2}P_{o}-P_{i}(t_{n})\right)\Theta_{+}(N_{\Omega_{i}}\left(t_{n}^{-}\right)-1)\cdot \\ \;\frac{\chi_{\Omega_{i}}(x)c\left(x,t_{n}^{-}\right)}{N_{\Omega_{i}}\left(t_{n}^{-}\right)}, \end{array}$$

with ***x*** ∈ Ω, $t_{n}=\frac{n}{\omega }$ ∊ 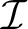, and *n* = 1, …, 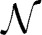
_*A*_. In the following, we explain the terms used in the above equations:

1. Given the interval 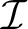, that represents the lapse of time during which the system is observed, the natural number 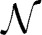
_*A*_ ∈ ℕ is the total number of times (modeled as points of 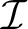) in which the action potential stimulates the depletion of vesicles. The generic instant of time *t*_*n*_ ∈ 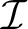, with *n* = 1, …, 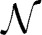
_*A*_, corresponds to the *n*th stimulus, and is defined by $t_{n}:=\frac{n}{\omega }$, with ω being the stimulation frequency.
2. *i* refers to the synapse number.
3. The mathematical definitions of the Dirac Delta δ, Heaviside distributions Θ and Θ_+_, and characteristic function χ_Ω_*i*__ are given in Section 3.9. The Dirac Delta δ (*t* − *t*_*n*_) restricts exocytosis to the time points *t*_*n*_ of action potentials; the characteristic function χ_Ω_*i*__ restricts the activity of the sink rate solely to the *i*th synaptic region. In the present setting, the Dirac Delta and the Heaviside distributions are dimensionless, while *h*(***x***, *t*^−^_*n*_) has units μm^−3^.
4. *P_o_* defines the release probability of vesicles at single synapses.
5. *N*_Ω_*i*__(*t*^−^_*n*_) − 1 is introduced to ensure that the Heaviside function Θ_+_ is equal to one, if there is at least one vesicle present at the corresponding release site. Therefore, in order to obtain the result Θ_+_(*N*_Ω_*i*__(*t*^−^_*n*_) − 1) = 1, the quantity *N*_Ω_*i*__(*t*^−^_*n*_) − 1 has to be non-negative.
6. A random generator produces values *P*_*i*_ between 0 and 1 for each synapse at every action potential. If *P*_*i*_ is lower than *P_o_*, a vesicle is exocytosed. This is regulated by the expression Θ(*P_o_* − *P*_*i*_(*t*_*n*_)) summed over all synapses *i*. Thus, in between action potentials the model describes a standard diffusion process.
7. Given a generic function of time, φ : 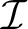 ↦ ℝ, the notation φ(*t*^−^_*n*_) stands for φ(*t*^−^_*n*_) = lim_*t* → *t*_*n*_^−^_ φ(*t*).

Moreover, the constant *a*_0_ shall be set equal to unity from here on. The sink term (8) is now treated in the following way. We conventionally set *t*_0_ ≡ 0 and *t*_
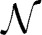
__*A*_ + 1 = *T*, and write the interval 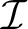 = [0, *T*] as 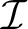 = ∪^
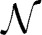
_*A*_ + 1^_*m* = 1_
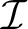
_*m*_, where

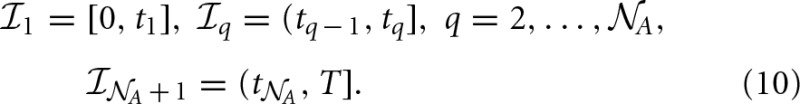



In each open interval (*t*_*m* − 1_, *t*_*m*_), *m* = 1, …, 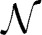
_*A*_ + 1, *f*_syn_(***x***, *t*) vanishes identically and, thus, the solution to Equation (4) represents pure diffusion. However, if at a given $t_{p}=\frac{p}{\omega }$ ∈ 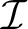, with *p* ∈ {1, …, 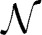
_*A*_}, the Heaviside functions featured in (9) are simultaneously different from zero for the synaptic region Ω_*i*_, the sink term introduces a jump in the value of concentration at *t*_*p*_, for all ***x*** ∈ Ω_*i*_. Hence, by employing the notation *c*(***x**, t*^±^_*p*_) = lim_*t* → *t*^±^_*p*__*c*(***x**, t*), we obtain

(11) $$c\left(x,t_{p}^{+}\right)=c\left(x,t_{p}^{-}\right)-\frac{c\left(x,t_{p}^{-}\right)}{N_{\Omega_{i}}\left(t_{p}^{-}\right)}.$$

The “updated” concentration *c*(***x**, t*^+^_*p*_) is treated as initial condition for the diffusion equation in the interval (*t*_*p*_, *t*_*p* + 1_).

We remark that, according to (11), only one vesicle is removed from the region Ω_*i*_ at *t*_*p*_. Indeed, by integrating (11) over Ω_*i*_, and recalling that *N*_Ω_*i*__(*t*^±^_*p*_) = ∫_Ω_*i*__ *c*(***x**, t*^±^_*p*_) d*v*, we obtain

(12) $$N_{\Omega_{i}}\left(t_{p}^{+}\right)=N_{\Omega_{i}}\left(t_{p}^{-}\right)-1.$$

We denote now by *c*_*n*_, with *n* = 1, …, 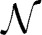
_*A*_, the restriction of the solution to Equation (4) to the interval (*t*_*n* − 1_, *t*_*n*_). By continuating each *c*_*n*_ to the time point *t*_*n*_, we set *c*_*n*_(***x**, t*_*n*_) ≡ *c*(***x**, t*^−^_*n*_), and define the number of vesicles contained in Ω_*i*_ at time *t*_*n*_ in the following way:

(13) $$N_{\Omega_{i}}(t_{n}):=\int_{\Omega_{i}}c_{n}(x,t_{n})\text{d}v=N_{\Omega_{i}}\left(t_{n}^{-}\right).$$

To evaluate the Heaviside function Θ_+_ of Equation (9), we use the number of vesicles *N*_Ω_*i*__(*t*_*n*_), determined in Equation (13).

More generally, however, for a given ***x*** ∈ Ω, the jump in the vesicle concentration at time *t*_*n*_, with *n* = 1, …, 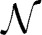
_*A*_, is given by

(14) $$\begin{array}{l} c\left(x,t_{n}^{+}\right)=c\left(x,t_{n}^{-}\right)-\sum_{i\;=\;1}^{S}\Theta \left(P_{o}-P_{i}(t_{n})\right)\Theta_{+}(N_{\Omega_{i}}\left(t_{n}^{-}\right)-1)\\ \;\frac{\chi_{\Omega_{i}}(x)c\left(x,t_{n}^{-}\right)}{N_{\Omega_{i}}\left(t_{n}^{-}\right)}, \end{array}$$

with ***x*** ∈ Ω. The concentration *c*(***x**, t*^+^_*n*_) of Equation (14) is the “initial condition” that has to be used for solving Equation (4) in the interval (*t*_*n*_, *t*_*n* + 1_). We remark that, when the vesicle concentration inside the synaptic regions is such that it is safely approximated by its mean value, i.e., *N*_Ω_*i*__/Vol(Ω_*i*_), for all *i* = 1, …, *S*, Equation (11) becomes

(15) $$c\left(x,t_{p}^{+}\right)=c\left(x,t_{p}^{-}\right)-\frac{1}{\text{Vol}(\Omega_{i})}.$$

Consequently, Equation (14) can be approximated as follows

(16) $$\begin{array}{l} c\left(x,t_{n}^{+}\right)=c\left(x,t_{n}^{-}\right)-\sum_{i\;=\;1}^{S}\Theta \left(P_{o}-P_{i}(t_{n})\right)\\ \;\Theta_{\;+}(N_{\Omega_{i}}(t_{n}^{-})-1)\frac{\chi_{\Omega_{i}}(x)}{\text{Vol}(\Omega_{i})}. \end{array}$$

We compute the variation of the overall number of vesicles contained in the bouton by integrating Equation (14) over Ω and using the properties of the characteristic functions (see Section 3.9), i.e.,

(17) $$N_{\Omega }\left(t_{n}^{+}\right)=N_{\Omega }\left(t_{n}^{-}\right)-\sum_{i\;=\;1}^{S}\Theta (P_{o}-P_{i}(t_{n}))\Theta_{+}(N_{\Omega_{i}}\left(t_{n}^{-}\right)-1).$$

We remark that the number of vesicles does not vary, i.e., *N*_Ω_(*t*^+^_*n*_) = *N*_Ω_(*t*^−^_*n*_), if either Θ(*P_o_* − *P*_*i*_(*t*_*n*_)) or Θ_+_(*N*_Ω_*i*__(*t*^−^_*n*_) − 1) is zero.

In order to determine the solution to Equation (4) in each interval 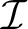
_*m*_, with *m* = 1, …, 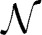
_*A*_ + 1, we consider the restriction *c*_*m*_ to 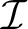
_*m*_, and discretize Equation (4) in time by using a first-order finite difference implicit Euler scheme. The result of this calculation reads

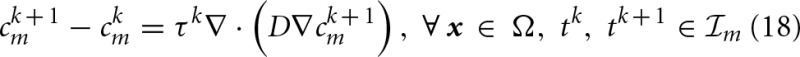



where *c*^ℓ^_*m*_(***x***) ≡ *c*_*m*_(***x**, t*^ℓ^), with ℓ = {*k, k* + 1}, denotes the concentration in ***x*** ∈ Ω at time *t*^ℓ^ ∈ 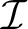
_*m*_, and τ^*k*^ = *t*^*k* + 1^ − *t*^*k*^ > 0 is the amplitude of the *k*th time step. Then, we solve Equation (18) by using the Finite Volume Method. For a detailed presentation of the solution method we refer to standard literature on Finite Volumes. In this paper, we sketch very briefly the procedure to compute *N*_Ω_*i*__(*t*^−^_*n*_) numerically. To this end, we firstly cover the entire computational domain with a finite element triangulation. The nodes of the triangulation are denoted by ***x***_*s*_, with *s* = 1, …, 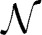
_*n*_ being the node index, and 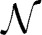
_*n*_ being the total number of nodes. The unknown of the problem, i.e., the vesicle concentration, is approximated by the sum 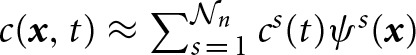, where *c*^*s*^(*t*) represents the time-varying value of concentration at the node ***x***_*s*_, whereas ψ^*s*^ is referred to as shape function. It is such that ψ^*s*^(***x***_*r*_) = δ^*s*^_*r*_, with δ^*s*^_*r*_ being the Kronecker-delta (it returns 1, if *r* = *s*, and it returns 0 otherwise). Secondly, we construct a *dual* grid by following the methods outlined in Frolkovic (1996). An “element” of the dual grid is referred to as finite volume. The finite volume associated with the node ***x***_*s*_ is denoted by *B*_*s*_ in this work, and is obtained by joining the barycenters of the elements sharing the node ***x***_*s*_ with the midpoints of the edges connecting ***x***_*s*_ with its neighboring nodes. The finite volumes *B*_*s*_, with *s* = 1, …, *N*_*n*_, cover the entire computational domain. For details the reader is referred to Frolkovic (1996). The whole procedure is applied also to the synaptic regions Ω_*i*_, with *i* = 1, …, *S*. Let then *B*_*ij*_ specifically denote the finite volume associated with the node ***x***_*ij*_ ∈ Ω_*i*_ of the discretization covering the synaptic region Ω_*i*_, with *j* = 0, …, *M*_*i*_ being the nodal index of the discretization of Ω_*i*_, and (*M*_*i*_ + 1) the total number of nodes of this discretization. Then, it holds that Vol(Ω_*i*_) = ⊔^*M*_*i*_^_*j* = 0_ Vol(*B*_*ij*_). Moreover, the number of vesicles *N*_Ω_*i*__(*t*^−^_*n*_) is approximated as follows

(19) $$\begin{array}{l} N_{\Omega_{i}}(t_{n}^{-})=\int_{\Omega_{i}}c_{n}\left(x,t_{n}^{-}\right)\text{d}v\\ \;\approx \sum_{j\;=\;0}^{M_{i}}c_{n}\left(x_{ij},t_{n}^{-}\right)\text{Vol}(B_{ij}),\;x_{ij}\in B_{ij}\subset \Omega_{i},\\ \;\forall \;j=0,\ldots ,M_{i}. \end{array}$$

Finally, by substituting Equation (19) into Equation (9), we obtain the discretized form of the expression *h*(***x***_*s*_, *t*^−^_*n*_):

(20) $$\begin{array}{l} h\left(x_{s},t_{n}^{-}\right)=\sum_{i\;=\;1}^{S}\Theta (P_{o}-P_{i}(t_{n}))\Theta_{+}(\sum_{j\;=\;0}^{M_{i}}c_{n}\left(x_{ij},t_{n}^{-}\right)\text{Vol}(B_{ij})-1)\\ \;\frac{\chi_{\Omega_{i}}(x_{s})c_{n}\left(x_{s},t_{n}^{-}\right)}{N_{\Omega_{i}}\left(t_{n}^{-}\right)}. \end{array}$$

### 3.5. Numerical methods

The model equations were discretized with finite volumes in space and an implicit Euler method in time. The resulting systems of linear equations were solved with numerical multigrid techniques using Gauss-Seidel pre- and post-smoothers and LU-decomposition as base solver. The resulting fine grid problems consisted of approximately 20,000–40,000 grid nodes and were solved on parallel architectures, the HERMIT Supercomputer at the HLRS Stuttgart and on the Cekon and Cesari cluster of the G-CSC.

### 3.6. Building the computational domain

Bouton morphologies were constructed with Blender and ProMesh and discretized with a tetrahedral meshing algorithm using TetGen, that is incorporated in ProMesh (Reiter and Wittum, 2014). Examples of the 3D geometries can be found in Figure 1.

### 3.7. Empirical laws for the *in silico* results

From the simulations performed in Figures 5A,C,E we derived fitted empirical laws that reproduce the qualitative features of the measured post-synaptic response. The three parameters that were varied are *bouton size, stimulation frequency* and *P_o_*. In the following we denote the number of exocytosed vesicle as *E*(*t*, ω) and *E*(*t, d*) respectively, the stimulation frequency as ω, the vesicle output probability as *P_o_*, the number of synapses as *S*, the bouton diameter as *d* and use characteristic time points *t*_*i*_ with *i* = 1, …, 6 for tuning the empirical laws. Note that the time-points *t*_2_, *t*_4_, *t*_6_ refer to the time-points in which the respective functions reach the value 0.01.

The characteristic time points originate from heuristic fits using logarithmic representations resp. projections in x and y space allowing for heuristic determination of approximating curves. The same holds true for the *E*(*t*, …) which are used in the following. All equations stated in the following are used in dimensionless form, yet for the sake of clarity we omit the procedure of making the equations dimensionless during the introduction of the following equations. In the following section we define θ(*x*) as

(21) $$\theta (x):=\text{ \{}\begin{array}{cc} 1 & x<0\\ 0 & x\geq 0 \end{array}$$

#### 3.7.1. Bouton size-variation law

Considering the bouton size to be variable we can investigate the effect of the bouton size on the postsynaptic response that was simulated in Figure 5C. From the *in silico* experiments, we derive the following empirical law with *S* = 7, 10, 14 for *d* = 2, 3, 5 μm respectively and *P_o_* := 6 %:

(22) $$\begin{array}{l} E(t,d)=P_{o}\cdot S\cdot (\theta \left(t-t_{1}(d)\right)+\theta (t_{1}(d)-t))\cdot \\ \;\exp\left(-\frac{\log(100\cdot P_{o}\cdot S)}{\left(t_{2}(d)-t_{1}(d)\right)}\cdot \left(t-t_{1}(d)\right)\right) \end{array}$$

with characteristic time points

(23) $$t_{1}(d)=\exp\left(\log(11.5)+\log\left(\frac{465}{11.5}\right)\cdot \frac{d}{5}\right)$$

(24) $$t_{2}(d)\;=\exp\left(\log(80)+\log\left(\frac{2900}{80}\right)\frac{d}{5}\right)$$

#### 3.7.2. Frequency-variation law

The effect of the stimulation frequency on the postsynaptic response was simulated in Figure 5E. From the *in silico* experiments, we derive the following empirical laws with *S*: = 10 and *P_o_*: = 6 %

(25) $$\begin{array}{l} E(t,\omega )=P_{o}\cdot S\cdot (\theta (t-t_{3}(\omega ))+\theta (t_{3}(\omega )-t)\cdot \\ \;\exp\left(-(t-t_{3}(\omega ))\frac{\log(10P_{o}S)}{\left(t_{4}(\omega )-t_{3}(\omega )\right)}\right)) \end{array}$$

with frequency-dependent characteristic time points

(26) $$t_{3}(\omega )\;=\exp\left(-0.027(2.7\omega -270)\right)$$

(27) $$t_{4}(\omega )\;=\exp\left(8.8-0.775\log(\omega )\right)$$

#### 3.7.3. *P_o_*-variation law

The effect of the vesicle output probability *P_o_* on the post-synaptic response was simulated in Figure 5A. From the *in silico* experiments, we derive the following empirical laws with *S*: = 10 and ω := 40 Hz

(28) $$E(t,P_{o})=\{\begin{array}{ll} \theta \left(P_{o}\cdot S-ℭ(t)\right)P_{o}\cdot S+\theta \left(ℭ(t)-P_{o}S\right)ℭ(t) & P_{o}\geq 10\%\\ \theta (t-t_{5})P_{o}\cdot S+\theta (t_{5}-t)P_{o}S & \\ \exp\left(\log(P_{o}\cdot S\cdot 100)\cdot \frac{\left(t-t_{5}(P_{o})\right)}{\left(t_{5}(P_{o})-t_{6}(P_{o})\right)}\right) & P_{o}<10\% \end{array}$$

with the characteristic time points

(29) $$t_{5}(P_{o})=0.3\;\cdot \;\exp\left(\frac{1}{\left(P_{o}+0.115\right)}\right)$$

(30) $$t_{6}(P_{o})=500\;\cdot \;\exp\left(\frac{2}{{\left(P_{o}\cdot 100\right)}^{1.2}}\right)$$

where

$$\begin{array}{l} ℭ(t):=\theta (t-3)\left(40^{\exp\left(-t^{0.9}\right)}+1\right)+\theta (3-t)\theta (t-18)\\ \;\cdot (1.07\cdot \exp(-0.25\;\cdot \;(t-3))+1.2)\\ \;+\theta (18-t)\theta (t-50)0.0102\;\cdot \;\exp(-0.012\;\cdot \;t+5)\\ \;+\;\theta (50-t)\exp\left(-t\;\cdot \;\frac{\log(100)}{560}+0.24\right) \end{array}$$

### 3.8. Norms for quantifying the simulation results

In order to compare the experimental results with the simulation results, we applied two norms to the data:

1. *Least-squares S*, defined as $S:=\frac{1}{n}\sqrt{\sum_{i\;=\;1}^{n}d_{i}^{2}}$
2. *L*_2_-*norm*, defined as $L_{2}:=\sqrt{\frac{d_{1}^{2}\frac{\left(p_{2}-p_{1}\right)}{2}+\sum_{i\;=\;2}^{n\;-\;1}d_{i}^{2}\frac{\left(p_{i\;+\;1}-p_{i-1}\right)}{2}+d_{n}^{2}\frac{\left(p_{n}-p_{n\;-\;1}\right)}{2}}{\left(p_{n}-p_{1}\right)}}$

where $d_{i}=\left|\frac{\left(s_{i}-\;e_{i}\right)}{\left(s_{i}+\;e_{i}\right)}\right|$, with *e*_*i*_ being the experimental data points and *s*_*i*_ the simulated data points. *p*_*i*_ denote the stimulus number.

### 3.9. Summary of the mathematical expressions used in the model

The symbols δ, Θ and χ_Ω_*i*__ denote, respectively, the Dirac's Delta distribution, the Heaviside function and the characteristic function associated with the set Ω_*i*_. The symbol Θ_+_ is used to modify the definition of Θ, as explained below.

Given a set *V* ⊂ ℝ^*d*^, with ℝ^*d*^ being the *d*-dimensional Euclidean space, the function χ_*V*_ : ℝ^*d*^ → {0, 1} is said to be the characteristic function associated with *V*. It is defined as follows

(31) $$\chi_{V}(x)=\{\begin{array}{ll} 1, & \text{if}\;x\in V,\\ 0, & \text{otherwise}. \end{array}$$

Let *V* ⊂ Ω be a subset of Ω and let *f* : Ω → ℝ be an integrable function over *V*. Then, the following identity holds true

(32) $$\int_{\Omega }\chi_{V}(x)f(x)\text{d}v=\int_{V}f_{|V}(x)\text{d}v,$$

where *f*_|*V*_ represents the restriction of *f* to *V*. In particular, if *f* is everywhere equal to unity, then it follows that

(33) $$\int_{\Omega }\chi_{V}(x)\text{d}v=\int_{V}\text{d}v=\text{Vol}(V).$$

The Heaviside function, Θ, is defined as

(34) $$\Theta (\xi )=\{\begin{array}{ll} 1, & \text{if}\;\xi >0,\\ 0, & \text{if}\;\xi <0. \end{array}$$

If the function is required to return unity also for ξ = 0, then the definition (34) is extended as follows

(35) $$\Theta_{+}(\xi )=\{\begin{array}{ll} 1, & \text{if}\;\xi \geq 0,\\ 0, & \text{if}\;\xi <0. \end{array}$$

The Dirac Delta is a distribution. Given an arbitrary function ϕ : ℝ → ℝ, the Dirac Delta satisfies the important property

(36) $$\int_{\mathbb{R}}\delta (s-t)\phi (s)\text{d}s=\phi (t),$$

if ϕ is continuous at *t* ∈ ℝ.

## 4. Discussion

In this study we have developed a three-dimensional model of presynaptic vesicle dynamics to assess the physiological meaning of experimentally rather inaccessible bouton parameters like the bouton size with a given release site density, vesicle diffusion and the vesicle release probability. In combination with the stimulation frequency and an associated release of vesicles these parameters represent simplified but fundamental bouton output functions that should allow a systematic structure/function analysis of two very similar classes of glutamatergic boutons.

### 4.1. A three-dimensional model of local vesicle dynamics

In addition to state of the art modeling approaches to synaptic or neuronal functions we used methods that represent the three-dimensional morphology and biological processes derived from physical principles. Electrical signaling of neurons is often modeled as a one-dimensional process using the cable equation and compartmental one-dimensional space discretization (Hines and Carnevale, 2001). There are few approaches in cellular modeling that make use of 2D or 3D modeling techniques (Smart and McCammon, 1998; Ross et al., 2000; Meinrenken et al., 2002; Tai et al., 2003; Marpeau et al., 2009; Bielecki et al., 2010). Most commonly, stochastic models are employed to address the high complexity of three-dimensional simulations. A standard approach to *in silico* studies of presynaptic vesicle dynamics is to model the transition of vesicles between defined states with preconfigured transition-rate constants (Pan and Zucker, 2009), thus reducing the three-dimensional process of vesicle motion to a time-dependent process (zero-dimensional in space). Considering the underlying question of our study presented here, we needed to develop a model that takes into account the spatial dynamics of vesicles in 3D.

Modeling neurobiological systems requires incorporating the physical laws to which these systems abide. The use of deterministic models derived from continuum mechanics theory allows us to describe spatio-temporal vesicles dynamics based on physical laws which allows for a much more profound understanding of the problem compared to the use of heuristic models which are afterwards fitted to data. The parameters used for computations were taken either from the literature or EM sections and no parameter fitting was used. Combined with a three-dimensional representation of the morphology using unstructured grids, in this case different types of presynaptic boutons, this approach can answer questions regarding structure and function relationships. The equations are numerically approximated by a finite volumes approach and solved on parallel computers. The added mathematical techniques allowed us to investigate the interplay between structure and function at presynaptic terminals in a highly realistic manner. In addition to the three-dimensional representation of the underlying biophysics and three-dimensional morphology, our approach enables us to continually refine the model by including more of the systems properties, such as vesicle recycling, calcium dynamics and others depending on the current focus.

### 4.2. Vesicle diffusion limits the availability of preexisting vesicles at active zones

Our simulations modeled vesicle motility during stimulation as a simple undirected diffusion process. These simulations matched our experimental results (Figures 1E,F, 3, 4) suggesting that vesicle diffusion describes the motility of preexisting vesicles during periods of high frequency stimulation quite accurately. However, there is a debate whether or not active and directional transport mechanisms based on, e.g., the Myosin/Actin system are directly contributing to synaptic transmission (e.g., Ryan, 1999; Jordan et al., 2005; Tokuoka and Goda, 2006; Seabrooke et al., 2010; Kisiel et al., 2011; Seabrooke and Stewart, 2011). In particular in the Drosophila NMJ system recent evidence has implicated non-muscle myosin II and the unconventional myosin VI in mobilizing synaptic vesicles during synaptic transmission (Seabrooke et al., 2010; Kisiel et al., 2011; Seabrooke and Stewart, 2011). In our study we focused on high frequency synaptic transmission in the absence of vesicle recycling and hence analyzed exclusively the motility of preexisting mature vesicles during extended periods of naturally occurring firing frequencies of 10–80 Hz. Under these conditions the modeled slow vesicle diffusion was sufficient to rather accurately mimic our eEJP-depletion experiments (Figures 3, 4) and they showed no obvious requirement for the incorporation of a faster vesicular transport mechanism. However, our results also showed that vesicle recycling plays a major role during prolonged high frequency stimulation as indicated by the much slower eEJP-depletion kinetics in the absence of BafA1 (Figure 3D). It seems therefore possible that fast active transport mechanisms are primarily involved in vesicle recycling processes that rapidly replenish active zones with vesicles during periods of prolonged stimulation. In contrast, preexisting vesicles that may for example be part of the reserve pool of vesicles and that have been assayed in this study seem to be recruited to active zones primarily by diffusion. Apparently, the glutamatergic boutons of Drosophila NMJs use both active and passive vesicular transport mechanisms. Whether and how they interact and what their relative contributions to the rather short natural activity patterns (Figure 6C) are has to be analyzed in future studies.

Based on our finding that vesicle diffusion is the primary factor limiting the replenishment of active zones with preexisting vesicles, we have assayed its effects on synaptic performance under various conditions. Depending on the activity of a given release site, which was determined by its release probability, its stimulation frequency and the typical number of stimuli per pattern, the local vesicle concentration dropped rapidly resulting in individual transmission failures and hence in a fast decline of the total bouton output (Figures 5A,B,E,F). The size of a bouton affects these parameters indirectly by offering, e.g., proportionally more active zones in larger boutons and simultaneously a larger functional reservoir of vesicles resulting in this example in a larger and more enduring bouton output, respectively (Figures 5C,D). Reliable transmission of a given activity pattern therefore depended strongly on the relative settings of these parameters. Based on these simulations we predict that a small type Is bouton with its smaller number of rather high *P_o_*-release sites is inappropriate for the transmission of long-lasting high stimulation frequencies (Figure 5) and should quickly become diffusion-limited (white patches in right bouton of Figure 6B). This effect is governed by both bouton size and the corresponding number of release sites. In contrast, a large type Ib bouton with its larger number of low *P_o_*-release sites is likely to be better adapted to transmit such a pattern (Figure 5; left bouton in Figure 6B). Indeed, many experimental high frequency stimulation paradigms, in which both motor neurons were fired simultaneously by a common stimulation electrode showed a depression of early compound eEJP-amplitudes before reaching plateau values (Delgado et al., 2000).

Our predictions now revealed that the observed eEJP-depression in HFS-trains is likely due to an initial dominance and rapid decay of the Is output while the Ib output maintains its reliable transmission (Figure 6A). It thus seems obvious that *in vivo* Is and Ib motor neurons can not be fired in a synchronous manner. Instead, it is more likely that the type-Is motor neuron is either fired with a low stimulus frequency or with only few stimuli, or both. Type Ib motor neurons are, in contrast, capable of reliably transmitting longer lasting high frequency patterns. We found that the natural firing activities of both motor neurons (Figures 6C,D) were strikingly consistent with the above predictions indicating that motor neuron firing patterns and NMJ-bouton properties are well-adapted features of this communication pathway.

### 4.3. The prevailing firing pattern shapes bouton properties

It has been reported in several central and peripheral systems including the *Drosophila* NMJs that long-lasting changes in the neuronal firing activity can change the physiological and morphological properties of the corresponding output synapses (Mollgaard et al., 1971; Hubel et al., 1977; Lnenicka and Atwood, 1989; Lnenicka et al., 2003; Sigrist et al., 2003). From these observations the concept emerges that the prevailing firing pattern of a neuron shapes the properties of its output synapses to ensure reliable transmission of its typical patterns. The simulations performed in this study revealed several tools a neuron can use to tune its output performance to its specific needs (Figure 7).

Under these conditions the developing bouton has to find an economic and still effective/reliable compromise between its size, i.e., the number of vesicles and release sites and their release probabilities (Figure 7B). A sustained high-frequency pattern would require a relatively large bouton with low release probability release sites. In contrast, if the prevailing activity pattern consists of only few APs at a rather low frequency an economic realization could be a smaller bouton with high-probability release sites. For a developing *Drosophila* NMJ these are the equivalents of type Ib and type Is boutons, respectively (Figures 1A, 7C). Early in larval development, when the innervated muscles are very small only few boutons of each type are required to ensure the transmission of the motor pattern and an efficient excitation-contraction coupling. The larger the muscle grows the less efficient excitation-contraction coupling becomes, requiring the proportional addition of more boutons of both types (Schuster et al., 1996). In addition to this developmental change of boutons, larval NMJs can undergo an experience-dependent bouton adaption (Sigrist et al., 2003). The morphology of a mature NMJ (Figure 7C) therefore likely represents a cumulative readout of its prevailing firing pattern throughout development.

### 4.4. Concluding remarks

Our three-dimensional functional model of multi-release site boutons allowed us to dissect the physiological meaning of experimentally inaccessible parameters like the bouton size, vesicle diffusion and the release probability. Based on the simulations with the three-dimensional model, we were able to derive an empirical and analytical description of the complex system subject to variations in bouton size, release probability and stimulation frequency. Our model sets the framework for a theoretical reconstruction and functional dissection of further realistic parameters such as vesicle recycling, the membrane potential and its dynamics during stimulation, the implementation of voltage-dependent calcium channels and intracellular calcium transients etc. Many of these parameters are experimentally difficult to access. Their implementation into a realistic theoretical model will therefore enable insights that have previously been impossible.

### Conflict of interest statement

The authors declare that the research was conducted in the absence of any commercial or financial relationships that could be construed as a potential conflict of interest.
