# Supplementary figures and images for: Synaptic bouton properties are tuned to best fit the prevailing firing pattern
Source: Front Comput Neurosci. 2014 Sep 9;8:101. doi: 10.3389/fncom.2014.00101 (PMC4158995; doi:10.3389/fncom.2014.00101)

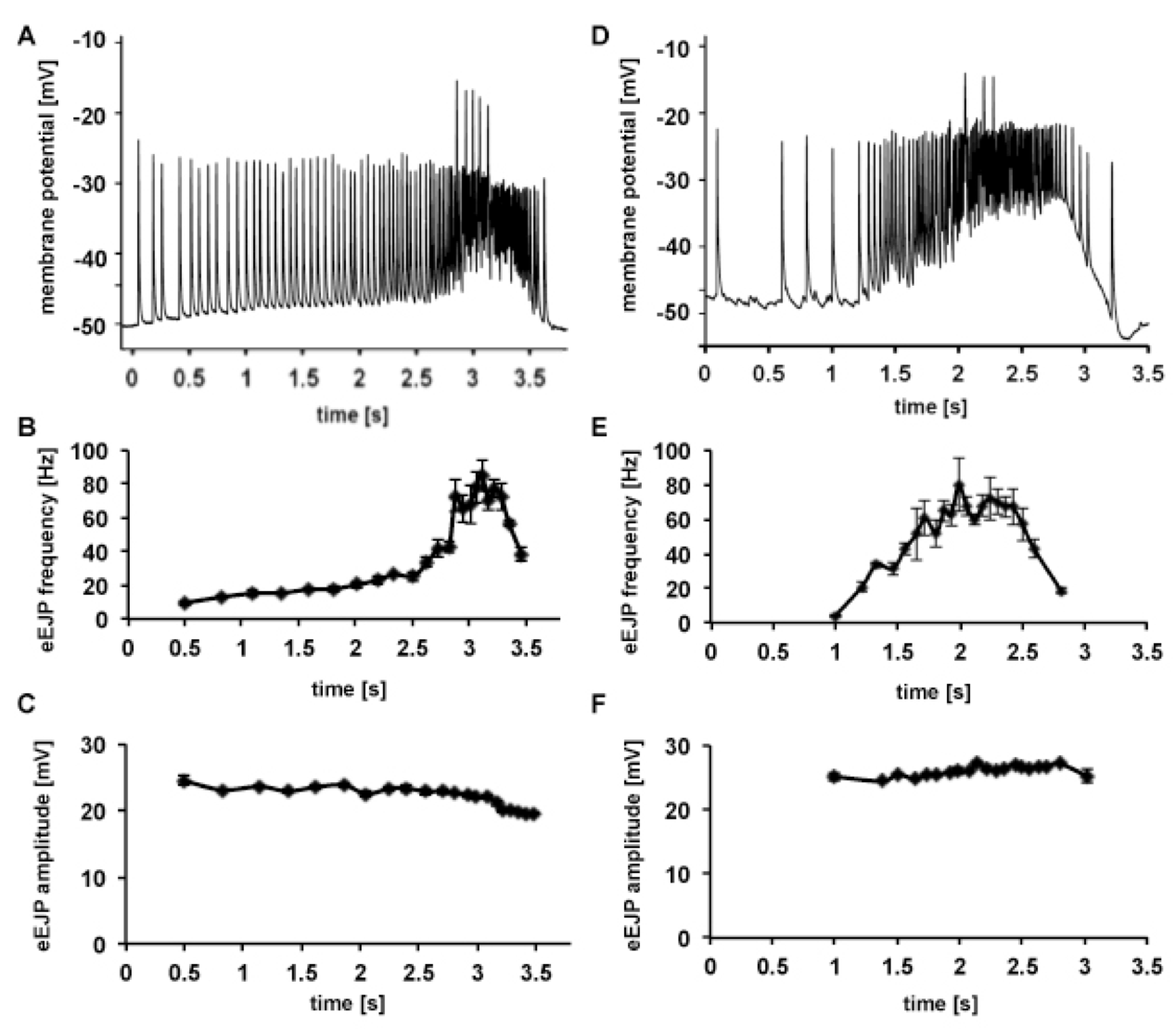

Supplement: Supplementary file 2 [file Image1.TIFF]
